# Neuroanatomical Alterations in High-Functioning Adults with Autism Spectrum Disorder

**DOI:** 10.3389/fnins.2016.00237

**Published:** 2016-06-02

**Authors:** Tehila Eilam-Stock, Tingting Wu, Alfredo Spagna, Laura J. Egan, Jin Fan

**Affiliations:** ^1^Department of Psychiatry, Icahn School of Medicine at Mount SinaiNew York, NY, USA; ^2^Department of Psychology, Queens College, City University of New YorkFlushing, NY, USA; ^3^The Graduate Center, City University of New YorkNew York, NY, USA; ^4^Department of Neuroscience, Icahn School of Medicine at Mount SinaiNew York, NY, USA; ^5^Friedman Brain Institute, Icahn School of Medicine at Mount SinaiNew York, NY, USA

**Keywords:** autism, voxel-based morphometry, gray matter volume, autism brain imaging data exchange, ABIDE

## Abstract

Autism spectrum disorder (ASD) is a pervasive neurodevelopmental condition, affecting cognition and behavior throughout the life span. With recent advances in neuroimaging techniques and analytical approaches, a considerable effort has been directed toward identifying the neuroanatomical underpinnings of ASD. While gray-matter abnormalities have been found throughout cortical, subcortical, and cerebellar regions of affected individuals, there is currently little consistency across findings, partly due to small sample-sizes and great heterogeneity among participants in previous studies. Here, we report voxel-based morphometry of structural magnetic resonance images in a relatively large sample of high-functioning adults with ASD (*n* = 66) and matched typically-developing controls (*n* = 66) drawn from multiple studies. We found decreased gray-matter volume in posterior brain regions, including the posterior hippocampus and cuneus, as well as increased gray-matter volume in frontal brain regions, including the medial prefrontal cortex, superior and inferior frontal gyri, and middle temporal gyrus in individuals with ASD. We discuss our results in relation to findings obtained in previous studies, as well as their potential clinical implications.

## Introduction

Autism spectrum disorder (ASD) is a neurodevelopmental condition characterized by abnormal social interactions and communication, repetitive behaviors, restricted interests, and atypical sensory processing (American Psychiatric Association, 2013). Advances in neuroimaging techniques and analyses over the past two decades have led to a burgeoning of structural studies aimed toward identifying the neuroanatomical underpinnings of ASD. Overall, findings suggest a complex neurodevelopmental trajectory, characterized by an early brain overgrowth (Courchesne et al., 2003; Zielinski et al., 2014; Zwaigenbaum et al., 2014), followed by arrested growth later in childhood and early adolescence (Courchesne et al., 2001; Mak-Fan et al., 2012), and accelerated neural atrophy later in adulthood (Courchesne et al., 2011; Lange et al., 2015). While studies were able to localize the neuroanatomical alterations in ASD to specific brain regions (Carper and Courchesne, 2005; Schumann et al., 2010; Scheel et al., 2011; Zielinski et al., 2014; Dierker et al., 2015; Libero et al., 2015), structures (Stanfield et al., 2008; Schumann et al., 2009; Via et al., 2011; Nickl-Jockschat et al., 2012; Maier et al., 2015) and networks (Ameis et al., 2011; Barttfeld et al., 2011; Solso et al., 2015), reports have been largely inconsistent.

The inconsistency in neuroanatomical findings of previous studies may have stemmed from several factors, including differences in methodology, data acquisition, analytical approaches, clinical and demographic characteristics of the samples, as well as small sample-sizes. As ASD is a complex condition with multiple etiologies, risk factors, and diverse clinical manifestations (Amaral et al., 2008; Ecker et al., 2013b; Chen et al., 2015), there is an inherent variability among individuals with ASD that is likely related to variations in neuroanatomical abnormalities. Indeed, ASD is linked to a great variety of gene mutations, each of which has the potential to affect neural development through different pathways and in different ways, including gene transcription, expression and regulation, protein synthesis and translation, synaptic formation and function, as well as cell migration (Persico and Bourgeron, 2006; Sahin and Sur, 2015). The clinical manifestation of ASD symptoms can also vary between affected individuals (Amaral et al., 2008), and there is an ongoing debate among scientists and clinicians regarding the inclusion of previously diagnostically-segregated groups (e.g., Asperger's syndrome vs. autism) under the unifying umbrella of the new guidelines for ASD diagnosis (Mcalonan et al., 2008; Toal et al., 2010; Mandy et al., 2012). In addition, intelligence quotient (IQ) scores vary significantly between individuals with ASD, with intellectual disability in the majority of affected individuals, but average or above-average scores in the high-functioning end of the spectrum (Toal et al., 2010).

In order to address the inconsistency in neuroanatomical reports of ASD, recent studies have used meta-analytic approaches (Cauda et al., 2011; Duerden et al., 2012; DeRamus and Kana, 2015), larger sample-sizes (Toal et al., 2010; Ecker et al., 2012; Haar et al., 2014; Itahashi et al., 2015; Sussman et al., 2015), and stricter inclusion criteria according to age (Raznahan et al., 2009; Toal et al., 2010; Greimel et al., 2013), gender (Ecker et al., 2012; Itahashi et al., 2015), IQ (Ecker et al., 2012; Itahashi et al., 2015; Maier et al., 2015), and diagnosis (Mcalonan et al., 2008; Toal et al., 2010; Via et al., 2011). Multivariate classification techniques were also used in an attempt to better characterize the complex patterns of neuroanatomical alterations in ASD (Ecker et al., 2010a,b; Jiao et al., 2010; Uddin et al., 2011; Haar et al., 2014). Only a few studies, however, investigated brain anatomy in large, matched samples of high-functioning adults with ASD and typically-developing controls (TDC) (e.g., Ecker et al., 2012).

To mitigate issues of sample variability and inconsistent findings, we conducted a neuromorphometric study in a relatively large sample of high-functioning adults with ASD (*n* = 66) and gender, age, and IQ-matched TDC (*n* = 66). The samples were selected from the Autism Brain Imaging Data Exchange (ABIDE) database (Di Martino et al., 2014), and included data from ASD and TDC participants collected in a previous study from our lab as well. We used voxel-based morphometry (VBM) (Ashburner and Friston, 2000), an automated, unbiased, and conservative approach, to investigate alterations in regional gray-matter (GM) volume of individuals with ASD. We also examined the possible contributions of gender, age, and ASD symptom severity by including them as regressors in our model.

## Materials and methods

### Participants

The samples were selected from the ABIDE database (Di Martino et al., 2014), which is a multicenter database containing anatomical MRI scans, clinical measures, and demographic data from approximately 1000 participants, with age range of 6–65 years. The ABIDE database offers a non-precedent opportunity for investigating neuroanatomical alterations in large samples of individuals with ASD. The MRI data selected for this study were collected from ASD and TDC adult participants in three different sites: New York University Langone Medical Center (NYU), Social Brain Lab at the Research School of Behavioral and Cognitive Neurosciences, NeuroImaging Center, University Medical Center Groeningen and Netherlands Institute for Neurosciences (SBL), and Katholieke Universiteit Leuven (KUL). Only participants with T1 images and sites that provided a relatively large number of adult participants (at least 12 in each group) were included. Participants who could not be matched according to their demographic data were excluded. MRI data from a previous study conducted in our lab at the Icahn School of Medicine at Mount Sinai (ISMMS; Eilam-Stock et al., 2014) were also used. The total number of participants was 66 in the ASD group and 66 in the TDC group (NYU *n* = 19; SBL *n* = 15; KUL *n* = 14; ISMMS *n* = 18). Demographic information for the combined samples are shown in Table 1.

**Table 1:** **Demographic information**.

**Group**	***n***	**Age (years)**				**Gender**		**Full Scale IQ**			
		**Mean**	**SD**	**Max**	**Min**	***M***	***F***	**Mean**	**SD**	**Max**	**Min**
**TDC**											
Total	66	27	7	43	18	60	6	114	12	143	89
SBL	15	34	7	42	20	15		–	–	–	–
KUL	14	23	3	29	18	14		113	10	134	98
NYU	19	25	5	32	18	15	4	113	12	139	91
ISMMS	18	28	7	43	20	16	2	117	15	143	89
**ASD**											
Total	66	27	8	64	18	60	6	110	14	143	80
SBL	15	35	10	64	22	15		–	–	–	–
KUL	14	22	4	32	18	14		109	13	128	89
NYU	19	25	6	39	18	15	4	108	13	137	80
ISMMS	18	28	6	42	19	16	2	111	17	143	87

*TDC, typically-developed controls; ASD, autism spectrum disorder; SD, standard deviation; the two samples (TDC, ASD) did not differ in age [t_(130)_ = 0.20; p = 0.99] and in IQ [t_(113)_ = 1.70; p = 0.90]*.

Selected participants with ASD were all in the high-functioning end of the spectrum (IQ > 80), and received a DSM-IV-TR diagnosis of Autistic Disorder, Asperger's Disorder, or Pervasive Developmental Disorder Not-Otherwise-Specified. Detailed information regarding the diagnostic protocols for the ABIDE database at each site are publicly available on the ABIDE website (http://fcon_1000.projects.nitrc.org/indi/abide). After matching for gender, the ASD and TDC groups were matched on age across sites [*t*_(130)_ = 0.2; *p* = 0.99] and within each site separately [NYU *t*_(36)_ = 0.23; *p* = 0.81; SBL *t*_(28)_ = 0.40; *p* = 0.69; KUL *t*_(26)_ = −1.10; *p* = 0.28; ISMMS *t*_(34)_ = 0.18; *p* = 0.86]. The ASD and TDC groups were also matched on full score IQ (FSIQ) across sites [*t*_(113)_ = 1.7; *p* = 0.9] and within each site [NYU *t*_(36)_ = −1.16; *p* = 0.25; KUL *t*_(26)_ = −0.72; *p* = 0.48; ISMMS *t*_(34)_ = 1.25; *p* = 0.22], with the exception of participants from the SBL dataset for whom FSIQ scores were not available. Of note, however, all ASD and TDC participants from the SBL dataset were tested for FSIQ, and their scores were all within the normal range (http://fcon_1000.projects.nitrc.org/indi/abide/).

All sites contributing to the ABIDE database received approval from their local Institutional Review Boards for the acquisition of their data. In addition, all data retrieved from the ABIDE database are completely anonymous with no inclusion of protected health information, as required by the HIPAA guidelines (http://fcon_1000.projects.nitrc.org/indi/abide/). For the data acquired at ISMMS, all participants provided written informed consent, approved by the Institutional Review Board.

### Voxel-based morphometry analysis

To measure differences in GM volume between the ASD and TDC groups, we conducted VBM analyses using the VBM8 toolbox (http://dbm.neuro.uni-jena.de/vbm) and Statistical Parametric Mapping (SPM8, Welcome Trust Centre for Neuroimaging, University College London, UK) in MATLAB R2012b (Mathworks Inc., Sherborn, MA). First, all T1-weighted images were manually reoriented to the anterior commissure—posterior commissure plane to improve the coregistration of T1 images to the template. Then, each image was segmented into six tissue classes (i.e., GM, white matter, cerebrospinal fluid, bone, non-brain soft tissue, and air outside of the head and in nose, sinus, and ears) using the SPM standard tissue probability map (Mazziotta et al., 1995) with default parameters. Segmented GM images were spatially normalized to the “IXI500_MNI152” template, using the DARTEL algorithm (Ashburner, 2007) with default parameters. Non-linear warping for the effect of spatial normalization was corrected to generate these modulated normalized images, which represent relative volume after correcting for brain size. Each image was then smoothed using an 8-mm full width at half maximum Gaussian kernel.

A two-sample *t*-test was conducted for smoothed GM volume images from the ASD and TDC groups using a random-effect general linear model (GLM), with gender and age as nuisance regressors. Because the scans were taken at multiple sites which may have different MRI scanners and scanning protocols, an inherent variability may exist within the data. Therefore, we included an equal number of ASD and TDC participants within each site. We also used the locations as a dummy variable in our model. As suggested by the VBM8 manual, an absolute threshold mask of 0.1 was used for all the second-level analyses. To test the relationship between autism symptom severity and GM volume, we conducted an additional second-level GLM analysis for ASD participants, using their Autism Diagnostic Observation Schedule (ADOS) scores (Lord et al., 2000) as a regressor. Higher ADOS scores are indicative of increased ASD severity. Forty ASD participants for whom the ADOS scores were available (ABIDE *n* = 27; ISMMS *n* = 13) were selected from the original sample for this analysis. The significance level for the height of each voxel was set to *p* < 0.005 (uncorrected), with a contiguous-voxel extent threshold k > 17 voxels, to correct for multiple voxel comparisons. This threshold was estimated by using 10,000 Monte Carlo simulations with a customized Matlab program (Slotnick et al., 2003). The corrected a priori height threshold was *p* < 0.05.

## Results

### Between-group differences in gray-matter volume

**Figure 1:**
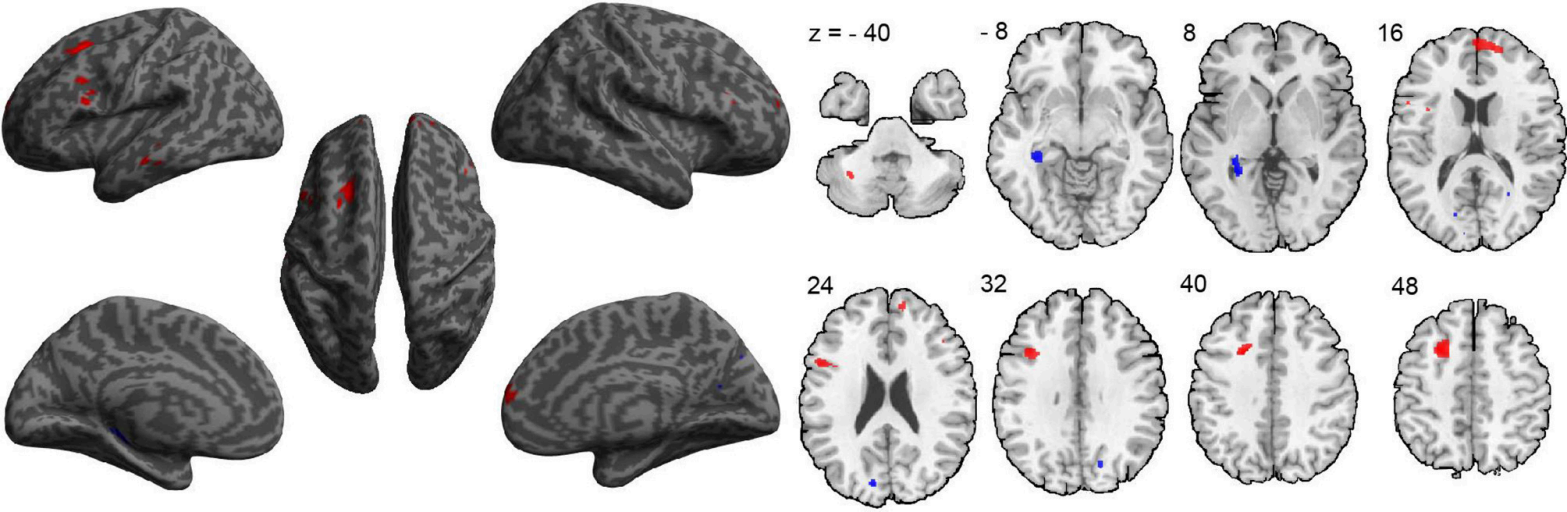
**Differences in gray-matter volume between the ASD and TDC groups**. Red indicates areas of increased gray-matter volume in ASD (ASD > TDC). Blue indicates areas of decreased gray-matter volume in ASD (ASD < TDC).

A between-group comparison of GM volume revealed increased volume in frontal, temporal, and cerebellar brain regions in the ASD group, compared to the TDC group. These regions included the medial prefrontal cortex (extending to the right), left superior frontal gyrus, left inferior frontal gyrus—pars opercularis (Broca's area), left inferior frontal gyrus—pars orbitalis, left middle temporal gyrus, and left cerebellum VIIb (Figure 1 and Table 2). In addition, compared to the TDC group, decreased GM volume in posterior brain regions in the ASD group was found, including the left posterior hippocampus and the cuneus bilaterally (Figure 1 and Table 2). These results remained consistent following an additional GLM analyses with age, gender, and site as nuisance regressors.

**Table 2:** **Brain regions with abnormal gray-matter volume in ASD**.

**Region**	**L/R**	**BA**	***x***	***y***	***z***	**T**	**Z**	**K**
**ASD** < **TDC**								
Posterior Hippocampus	L	36	−35	−36	−3	3.53	3.44	283
Cuneus	L	18/19	−12	−83	23	3.48	3.40	83
Cuneus	R	18	17	−72	32	2.82	2.77	31
**ASD** > **TDC**								
Superior frontal gyrus	L	8	−21	12	44	3.85	3.74	455
Superior frontal gyrus (medial)	R	10	2	54	11	3.50	3.41	634
Inferior frontal gyrus	L	44	−39	15	32	3.37	3.29	91
Inferior frontal gyrus	L	44	−51	11	26	3.35	3.27	196
Middle temporal gyrus	L	21	−62	−12	−14	3.12	3.06	86
Middle temporal gyrus	L	21	−59	−27	−12	3.06	3.00	35
Superior frontal gyrus (medial)	L	10	−18	62	21	2.97	2.91	28
Inferior frontal gyrus	R	45	44	29	26	2.92	2.87	18
Cerebellum VIIb	L		−33	−57	−41	2.91	2.86	24
Inferior frontal gyrus	L	47	−50	39	−15	2.87	2.82	34

*Height threshold: T = 2.72, p < 0.005, Extent threshold: k > 17*.

### Neuroanatomical correlations with ASD symptom severity

**Figure 2:**
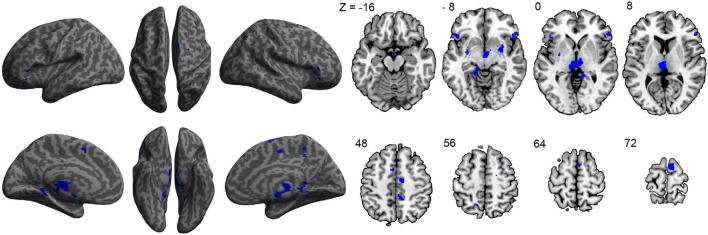
**Correlations between gray-matter volume and ASD symptom severity as indicated by the ADOS scores of each participant (***n*** = 40)**. All significant correlations found were negative and are indicated in blue.

To assess the relationship between ASD symptom severity and GM volume, ADOS scores of 40 participants with ASD were used as a regressor in our GLM model. Results revealed negative correlations between symptom severity and GM volume in the right superior frontal gyrus, left middle frontal gyrus, inferior frontal gyri—pars orbitalis bilaterally, restrosplenial cortex bilaterally, supplementary motor area bilaterally, right middle cingulate cortex, thalamus bilaterally, and putamen bilaterally (Figure 2 and Table 3), indicating that decreased GM volume in these regions is associated with more severe ASD symptoms. No significant positive correlations between symptom severity and GM volume were found.

**Table 3:** **Correlations between gray-matter volume and ASD symptom severity**.

**Region**	**L/R**	**BA**	***x***	***y***	***z***	**T**	**Z**	**K**
**NEGATIVE**								
Inferior frontal gyrus	R	47	54	24	−9	4.35	3.88	301
Inferior frontal gyrus	R	45	54	32	3	3.18	2.97	
Corpus mamillare	R		3	−12	−12	4.27	3.82	1342
Restrosplenial cortex	L	30	−15	−41	−6	3.67	3.36	
Thalamus	R		9	−18	−3	3.54	3.26	
Thalamus	L		6	−19	−3	3.33	3.09	
Mid cingulate cortex	R	6	14	−11	50	4.26	3.81	77
Restrosplenial cortex	R	30	8	−42	0	4.04	3.65	193
Inferior frontal gyrus	L	47	−47	23	−6	3.70	3.39	178
Supplementary motor area	R	6	6	0	74	3.69	3.38	198
Mid cingulate cortex	R	23	8	−39	48	3.24	3.02	139
Precuneus	L	7	−14	−59	56	3.22	3.00	19
Putamen	R		30	2	−9	3.10	2.90	143
Middle frontal gyrus	L	8/9	−32	36	44	3.07	2.87	20
Putamen	L		−30	−6	−6	2.97	2.79	95
Supplementary motor area	L	6	−9	9	51	2.96	2.78	79
Superior frontal gyrus	R	6	21	−6	59	2.90	2.73	25

*n = 40, Height threshold: T = 2.72, p < 0.005, Extent threshold: k > 17. Structures listed below clusters with a K-value were within that same cluster with different local maxima*.

## Discussion

### Gray-matter volume abnormalities along the anterior-posterior axis

Our results show a general pattern of increased GM volume in anterior brain regions and decreased GM volume in posterior brain regions in the ASD group, relative to TDC. A few theoretical accounts for the lobular specificity of neuroanatomical abnormalities in ASD across development have emerged, and may shed light on the differences in GM volume found in our study. Research on brain development in ASD across the lifespan has demonstrated a complex neurodevelopmental trajectory in affected individuals, characterized by an early brain overgrowth (Courchesne et al., 2003; Zielinski et al., 2014; Zwaigenbaum et al., 2014), followed by arrested growth later in childhood and early adolescence (Courchesne et al., 2001; Mak-Fan et al., 2012), and accelerated neural atrophy in adulthood (Courchesne et al., 2011; Lange et al., 2015). Studies in very young individuals with ASD (i.e., 2–4 years old) observed an increase of 5–12% in brain volume that was specifically localized to the frontal and temporal lobes (Carper et al., 2002; Redcay and Courchesne, 2005; Courchesne et al., 2007). This significant enlargement in anterior brain regions is reduced in older ages, though GM volume in these regions continues to be greater in ASD participants relative to TDC throughout development (1–3% increase) (Redcay and Courchesne, 2005; Courchesne et al., 2007). By contrast, the occipital lobe is not enlarged in young children with ASD (Carper et al., 2002; Courchesne et al., 2007). The occipital lobe is phylogenetically older than the frontal and temporal lobes, and its maturation occurs earlier in development (Ecker et al., 2013b); while the frontal and temporal lobes continue to develop throughout the first years of life, the occipital lobe does not change dramatically across the life span in typically developing individuals (Gogtay and Thompson, 2010).

Models of ASD suggest that the frontal and temporal enlargements that characterize early brain development in ASD may be a result of increased numbers of excitatory pyramidal neurons in these regions (Courchesne and Pierce, 2005a; Courchesne et al., 2007, 2011; Santos et al., 2011). This localized overgrowth may damage the local connectivity patterns within these regions, as well as the large-scale connectivity between these regions and the rest of the brain (Courchesne and Pierce, 2005b; Courchesne et al., 2007; Geschwind and Levitt, 2007; Ecker et al., 2013b; Chen et al., 2015). In the typically-developing brain, the connectivity patterns that develop throughout the first years of life allow for the higher-level cognitive skills that develop at the same time, including socio-emotional skills, language, and executive functions (Akshoomoff et al., 2002; Courchesne et al., 2007). Thus, it is reasonable to hypothesize that the aberrant connectivity patterns in ASD within the overgrowing frontal and temporal lobes, as well as between these regions and the rest of the brain, are at the core of the cognitive and behavioral deficits in ASD (Akshoomoff et al., 2002; Geschwind and Levitt, 2007). As individuals with ASD reach adulthood, processes of accelerated neuronal atrophy take place throughout the brain (Courchesne et al., 2011; Lange et al., 2015), perhaps to compensate for the early overgrowth in these individuals. The early localized enlargement of the anterior brain in ASD, together with the later broader neuronal atrophy in these individuals may, therefore, account for both our and others (e.g., Ecker et al., 2012) findings in adult ASD samples. The accelerated atrophy in adulthood may cause a GM volume decrease in both anterior parts of the brain, which are still greater relative to TDC though to a lesser extent, as well as in the posterior brain (mainly in the occipital cortex), which is now reduced relative to TDC.

### Gray-matter volume abnormalities in cortical and sub-cortical brain regions

ASD is a complex disorder with multiple symptoms affecting both high-level (e.g., socio-emotional processing, self-referential processing, language) and low-level (e.g., sensory processing) functions. It is not surprising, therefore, that the extent of neuroanatomical alterations found in our study, as well as in previous empirical investigations, encompasses regions, structures, and neural networks throughout the brain. A hallmark of ASD is abnormal socio-emotional processing, including deficits in theory of mind (i.e., the ability to understand other's beliefs, intentions and perspectives; Baron-Cohen et al., 1985; Baron-Cohen, 2000; Pilowsky et al., 2000), affective evaluations (Hill et al., 2004; Dapretto et al., 2006), and empathy (Minio-Paluello et al., 2009; Fan et al., 2013; Hadjikhani et al., 2014; Gu et al., 2015). Theory of mind relies on several neural regions, including the medial prefrontal cortex, lateral orbitofrontal cortex, middle frontal gyrus, superior temporal gyrus, temporal pole, temporoparietal junction, and cuneus (Frith and Frith, 1999; Gallagher and Frith, 2003; Saxe and Kanwisher, 2003; Amodio and Frith, 2006; Völlm et al., 2006). In our study we found GM abnormalities in the medial prefrontal cortex (increased GM volume) and cuneus (decreased GM volume). We also found negative correlations between GM volume in the middle frontal gyrus and ASD symptom severity. These structural abnormalities may be related, therefore, to the commonly seen theory of mind deficits in individuals with ASD. Our finding of increased GM volume in the medial prefrontal cortex in ASD may also explain the emotional evaluation difficulties commonly seen in this disorder, as this region plays a role in that domain as well (Phan et al., 2002; Harris et al., 2007; Etkin et al., 2011).

Our results of GM abnormalities in the inferior frontal gyrus, but not the ventromedial prefrontal cortex, may be related to a specific deficit in emotional empathy (e.g., feeling another person's pain) but preserved cognitive empathy (e.g., understanding that another person is in pain) in ASD (Minio-Paluello et al., 2009; Fan et al., 2013; Hadjikhani et al., 2014). Indeed, a recent lesion study demonstrated an anatomical dissociation between the cognitive and emotional components of empathy, such that the ventromedial prefrontal cortex is necessary for cognitive empathy, while the inferior frontal gyrus is essential for emotional empathy (Shamay-Tsoory et al., 2009). Additionally, in a functional MRI study investigating brain regions associated with empathy for pain, we found abnormal brain activation in the inferior frontal gyrus in ASD, with no group differences in ventromedial prefrontal cortex activation (Gu et al., 2015).

Our findings also point to several GM abnormalities in ASD that may be related to limited self-referential processing (Lombardo et al., 2007, 2010; Uddin, 2011) and autobiographical memory (Bowler et al., 2000; Crane and Goddard, 2008; Lind and Bowler, 2010) in this disorder. Studies that investigated the neural substrates of self-referential processing in typically-developing samples found that these processes activate a set of regions along the medial axis of the brain, commonly termed cortical midline structures (Northoff et al., 2006), including the medial prefrontal cortex/pregenual anterior cingulate cortex, the dorsomedial prefrontal cortex/middle cingulate cortex, and the precuneus/posterior cingulate cortex (Kelley et al., 2002; Northoff et al., 2006; Lombardo et al., 2010). The left inferior frontal gyrus was also found to be activated during self-related judgments (Kelley et al., 2002). In addition, the posterior hippocampus is involved in the storage and retrieval of autobiographical memories (Fernández et al., 1998; Kim, 2015). Our results of increased GM volume in the medial prefrontal cortex and the left inferior frontal gyrus, decreased GM volume in the posterior hippocampus, and negative correlations between GM volume in the middle cingulate cortex and precuneus and ASD symptom severity, may be related, therefore, to aberrant self-referential processing and autobiographical memory in individuals with ASD.

The increased GM volume in the left inferior frontal gyrus and left middle temporal gyrus in the ASD group in the present study may be related to altered language functions in affected individuals, especially in the semantics domain. Although language abilities vary greatly across the ASD spectrum, ranging from a severe language delay to normal language development, there is empirical evidence suggesting that semantic processing is compromised even in high-functioning individuals with ASD who do not exhibit any language delay (Harris et al., 2006; Kamio et al., 2007). High-functioning adults with ASD also showed significantly reduced activation in the left inferior frontal gyrus (Broca's area) during semantic processing (Harris et al., 2006). Indeed, the left inferior frontal gyrus, together with the left middle temporal gyrus, is involved in semantic processing in the typically-developing brain (Goel and Dolan, 2001; Visser et al., 2012). In our study, both of these regions were identified as areas of increased GM volume in ASD, which may serve as neuroanatomical substrates for the abnormal semantic processing in this disorder.

Although we did not find GM alterations in the thalamus in ASD, we did find a significant negative correlation between thalamic GM volume and ASD symptom severity. In addition, we observed decreased GM volume in the cuneus in the ASD group. The thalamus is a main hub for sensory processing across modalities, and it can affect sensory perception by integrating and relaying feedforward and feedback information between the sensory cortices and higher-order cortical regions (e.g., frontal lobe; Alitto and Usrey, 2003; Cudeiro and Sillito, 2006; Briggs and Usrey, 2008). The cuneus is a secondary visual area which may play a role in modulation of visual processing (Vanni et al., 2001). Abnormal sensory processing (both hyper-and-hypo-sensitivity) have been extensively documented in the ASD literature, especially in the visual modality (Behrmann et al., 2006; Vandenbroucke et al., 2008), and are now included in the ASD diagnostic criteria in the diagnostic and statistical manual of mental disorders (DSM-5) (American Psychiatric Association, 2013). Together, these findings may be related to the abnormal sensory processing commonly seen in individuals with ASD.

## Limitations

Although our results are consistent with some previous reports, they did not replicate other findings of GM alterations in adults with ASD. For example, in a study that specifically examined between-group differences in the amygdala and hippocampus in 30 high-functioning (IQ > 100) adults with ASD and gender, age, and IQ matched control participants (Maier et al., 2015), increased hippocampal volume bilaterally was found in ASD, opposite to our results. Yet, other studies failed to find any significant differences in GM volume between adults with ASD and TDC (Haar et al., 2014; Riedel et al., 2014; Riddle et al., 2016). This variability in structural findings may be due to relatively small sample sizes (Riedel et al., 2014; Maier et al., 2015) or differences in methodology and sample characteristics (Haar et al., 2014; Riddle et al., 2016). Thus, large-sample studies of different sub-groups within the ASD spectrum will likely promote a better characterization of neuroanatomical alterations that contribute to ASD symptomatology.

The current study took advantage of the relatively large sample of participants with ASD provided by the ABIDE database, and limited the inclusion criteria (i.e., high-functioning adults) to increase statistical power and reduce variability. However, as ASD is a complex condition with multiple contributing factors and etiologies, it is possible that our sample was not sufficiently homogeneous. While we attempted to control for different variables that may have contributed to the previously reported inconsistent findings, such as age and IQ, there are many other factors we did not take into account, such as genetic factors or clinical presentations. On the other hand, when using stricter inclusion criteria, the generalizability of the data is inevitably reduced. For example, it is possible that our findings represent neuroanatomical alterations in high-functioning adults with ASD only, and are less applicable to the majority of the ASD population, which has lower level of functioning and greater symptom severity. Studies with more individuals across the spectrum and higher severity of autism may shed a different light on the matter entirely. Future studies that continue to investigate neuroanatomy in large samples of affected individuals from different clinical and demographic subgroups, will, therefore, significantly contribute to our understanding of neuroanatomical alterations in individuals with ASD.

We measured GM volume using voxel-based morphometry, as this is one of the most informative and commonly used measures in the study of neuroanatomical abnormalities in clinical populations. However, other neuroanatomical measures were used in previous studies of ASD, which may also be useful indicators of structural abnormalities. These include measures of cortical folding and sulcal depth (Nordahl et al., 2007), cortical thickness (Hyde et al., 2010), cortical surface area (Ecker et al., 2013a), local gyrification index (Wallace et al., 2013), as well as diffusion tensor imaging for white-matter tract (Ameis et al., 2011; see Ecker et al., 2015 for review). Multivariate classification techniques were also recently used as a viable method for identifying complex patterns of neuroanatomical alterations in ASD (Ecker et al., 2010a,b; Jiao et al., 2010; Uddin et al., 2011; Haar et al., 2014). It would be valuable, therefore, to conduct studies with large samples that look at other structural measures as well.

## Clinical implications

Our study adds to the growing literature investigating neuroanatomical abnormalities in ASD. The research endeavor to characterize the profile of brain anatomy in ASD across development may have clinical implications, as it may facilitate identification of biomarkers for different subgroups within the ASD spectrum (Ecker et al., 2013b, 2015). While the behavioral markers of ASD have been extensively investigated and are relatively defined and agreed upon by researchers and clinicians, the neuroanatomical, neurofunctional and genetic profiles of ASD still warrant rigorous research. Once our knowledge of the different markers of ASD has been sufficiently advanced, the different pieces of the puzzle will come together to create a clear picture of this currently ill-understood disorder. This will allow for better diagnosis and treatment for ASD, which may be more specific to individuals or subgroups within the spectrum.

## Author contributions

All authors (TE, TW, AS, LE, and JF) contributed to data analysis and report writing.

## Funding

JF and AS were supported by the grant from Simons Foundation Autism Research Initiative (SFARI) 330704. JF was also supported by the National Institute of Mental Health of the National Institutes of Health under Award Number of and R21 MH083164. The content is solely the responsibility of the authors. The funders had no role in study design, data collection and analysis, decision to publish, or preparation of the manuscript.

### Conflict of interest statement

The authors declare that the research was conducted in the absence of any commercial or financial relationships that could be construed as a potential conflict of interest.
